# Neural network learning with photonics and for photonic circuit design

**DOI:** 10.1515/nanoph-2023-0123

**Published:** 2023-03-02

**Authors:** Daniel Brunner, Miguel C. Soriano, Shanhui Fan

**Affiliations:** FEMTO-ST/Optics Dept., UMR CNRS 6174, Univ. Franche-Comté, 15B avenue des Montboucons, 25030 Besançon Cedex, France; Instituto de Física Interdisciplinar y Sistemas Complejos, IFISC (UIB-CSIC), Campus Universitat de les Illes Balears, E-07122 Palma de Mallorca, Spain; Stanford University, Stanford, CA, USA

**Keywords:** artificial neural networks, nanophotonics, neural network assisted photonic design, photonic neural networks

This special issue covers works that lie at the interface between machine learning, spearheaded by the computing power of artificial neural networks (NN), and photonic technologies. In the past few years, there has been a renewed interest in this promising field due to a number of successful experimental demonstrations of advanced computing functionalities [1, 2] or the design of optimized nanophotonic devices [3, 4]. An example of the cross-fertilization from the combination of machine learning concepts and the advances in photonic fabrication using novel materials is the development of hardware accelerators for vector-matrix multiplication, which benefit from a software and hardware codesign [5].

Here, we have identified that current trends in the community can be conceptually divided in two distinct research directions. On the one hand, photonic systems and devices can serve as a hardware substrate that naturally suits the characteristic properties of artificial NN topologies [6]. Advantages brought by photonics in this context include the potential for parallelization, high-speed operation, and low power consumption. On the other hand, machine learning can aid in the design of photonic devices [7] or components [8] and accelerate the search for promising structures. Artificial NN can also assist in the processing of optically acquired data with the ultimate goal of adding new functionalities and enhancing performance [9].

Dinc et al. [10] provide a tomography-centered review about the involvement of NN in photonic circuit design, which provides a valuable general perspective to the scientific community and future candidate topologies of 3D optical design. The potential to simultaneously exploit the multiple physical dimensions of time, wavelength, and space for optical NN is reviewed in the context of recent advances of optical NNs by Bai et al. [11]. Brückerhoff-Plückelmann et al. [12] illustrate how charge accumulation can potentially be an ingredient for enabling large-scale photonic matrix processors; Gu et al. [13] provide a vision of 3D vertically integrated photonic NN based on vertical-cavity surface-emitting laser (VCSEL) arrays, while Buckley et al. [14] examine online learning paradigms for photonic NN, in which the machinery for training is built deeply into the hardware itself.

Li et al. [15] demonstrate how a periodically poled thin-film lithium niobate nanophotonic waveguide can be used to implement an ultra-fast and highly efficient optical neuron with a linear rectifying nonlinearity as activation function. By coupling a VCSEL laser to a resonant tunneling diode, Hejda et al. [16] demonstrate how to realize an optoelectronic excitable neuron. Hasegawa et al. [17] investigate parallel and deep all-optical reservoir computing with delayed feedback-coupled semiconductor lasers by combining multiple reservoirs in potentially hybrid configurations. Miri et al. [18] show how to harness the collective behavior of laser networks for storing and retrieving a large number of phase patterns, where nonreciprocal coupling is shown to be important for resource efficiency.

How to realize learning of multiple tasks in deep diffractive NN by leveraging multiple wavelengths is investigated and demonstrated by Duan et al. [19]. Continuing with diffractive optical networks, Mengu et al. [20] implement permutation matrices and find indications that the capacity of the diffractive optical networks in approximating a given permutation operation increases proportional to the number of diffractive layers. The application of integrated photonic reservoirs in the context of 64-level quadrature amplitude modulated and directly detected signal in a Kramers–Kronig receiver fashion is reported by Masaad et al. [21]. Hülser et al. [22] create a link between high-level information processing metrics and the performance in a particular task, which they investigate based on a delay-reservoir architecture comprising coupled Stuart–Landau oscillators. How to use transfer learning in a novel way is shown by Bauwens et al. [23], who demonstrate that the concept can increase an analog hardware reservoir’s robustness against parameter drifts. Other important aspects such as limited system size in photonic circuits and a potentially lower bit precision is addressed by Giamougiannis et al. [24], who experimentally demonstrate a speed-optimized dynamic precision NN including tiled matrix multiplication. Basani et al. [25] investigate self-similar topologies of multiport interferometers based on integrated beamsplitter meshes following sine–cosine fractal decompositions of unitary matrices with respect to compactness and robustness against fabrication nonidealities. Finally, the optimization, i.e., training of such multiport interferometer meshes based on a drastically simplified yet high performant protocol is experimentally reported by Pai et al. [26].

Yesilyurt et al. [27] propose a NN-based inverse design technique enabled by a differentiable analytical solver that mitigates common fabrication challenges by including simulated systematic and random nonidealities. Multi-task topology optimization of photonic devices utilizing only low-spatial frequency components in the conjunction with deep NN is introduced and evaluated by Mao et al. [28].

In conclusion, this special issue provides reviews, perspectives, and a variety of research articles on the current themes emerging from the interplay between photonics and NN. We hope that this collection of articles covering the many relevant scientific and technological aspects will be of help of researchers entering the field as well as for those who are already established. Particularly in this fast moving field with the current rate of innovation, regular inventories of the trending research directions are of significant benefit. We would like to thank the Nanophotonics Publishing editor Dennis Couwenberg and publishing assistant Tara Dorrian for their constant support and technical assistance.
